# Nonsurgical Corrective Union of Osteoporotic Vertebral Fracture with Once-Weekly Teriparatide

**DOI:** 10.1155/2015/784360

**Published:** 2015-07-30

**Authors:** Naohisa Miyakoshi, Akira Horikawa, Yoichi Shimada

**Affiliations:** ^1^Department of Orthopedic Surgery, Akita University Graduate School of Medicine, 1-1-1 Hondo, Akita 010-8543, Japan; ^2^Igarashi Memorial Hospital, 1-17-23 Tsuchizakiminato-Chuo, Akita 011-0946, Japan

## Abstract

Osteoporotic vertebral fractures usually heal with kyphotic deformities with subsidence of the vertebral body when treated conservatively. Corrective vertebral union using only antiosteoporotic pharmacotherapy without surgical intervention has not been reported previously. An 81-year-old female with osteoporosis presented with symptomatic fresh L1 vertebral fracture with intravertebral cleft. Segmental vertebral kyphosis angle (VKA) at L1 was 20° at diagnosis. Once-weekly teriparatide administration, hospitalized rest, and application of a thoracolumbosacral orthosis alleviated symptoms within 2 months. Corrective union of the affected vertebra was obtained with these treatments. VKA at 2 months after injury was 8° (correction, 12°) and was maintained as of the latest follow-up at 7 months. Teriparatide has potent bone-forming effects and has thus been expected to enhance fracture healing. Based on the clinical experience of this case, teriparatide may have the potential to allow correction of unstable vertebral fractures without surgical intervention.

## 1. Introduction

Vertebral fractures are the most common fragility fractures in patients with osteoporosis [1]. Osteoporotic vertebral fractures usually heal with kyphotic deformities resulting from subsidence of the vertebral body when treated conservatively [2, 3]. To maintain vertebral height and prevent angulation, surgical treatments including kyphoplasties are indicated [4, 5]. However, we recently encountered a patient with severe osteoporosis in whom osteoporotic vertebral fracture was correctively healed with only teriparatide administration and hospitalization, with no need for surgical intervention. We describe herein our clinical experience with this patient. To the best of our knowledge, corrective vertebral union using antiosteoporotic pharmacotherapy without surgical intervention has not been reported previously in the English literature.

## 2. Case Presentation

**Figure 1:**
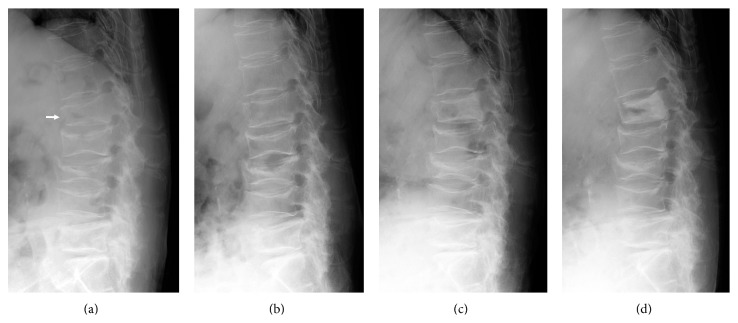
Plain lateral X-rays of the lumbar spine. (a) At the initial visit, the L1 vertebra shows a wedge-shaped deformity with a vertebral kyphosis angle (VKA) of 20° and a small intravertebral cleft (arrow). (b) At 2 weeks after injury, VKA of L1 is decreased (6°) compared to baseline, showing unstable vertebra. (c) At 2 months after injury, VKA of L1 is 8° with consolidation of the posterior wall. (d) At 7 months after injury, VKA of L1 is unchanged (8°) in the corrected position, although anterior intravertebral cleft remains.

An 81-year-old female with osteoporosis who had been treated with oral alendronate (35 mg/week) for 6 years presented with severe back pain after lifting a heavy object while cleaning the house. Plain lateral radiography of the spine showed multiple vertebral fractures from L1 to L5. Because the fractured L1 vertebral body showed an anterior cortical defect and small intravertebral cleft, the lesion at L1 was diagnosed as fresh vertebral fracture (Figure 1(a)). Magnetic resonance imaging of the spine confirmed that fresh fracture was only present at L1, and L2–L5 showed old prevalent fractures (data not shown). Segmental vertebral kyphosis angle (VKA) at L1, measured as the angle between cranial and caudal endplates [2], was 20° at diagnosis. A soft brace (thoracolumbosacral orthosis (TLSO)) was applied at diagnosis and she was instructed to rest in her house as much as possible. Alendronate was stopped, and the patient started to receive weekly subcutaneous injections of teriparatide (56.5 *μ*g of Teribone; Asahi Kasei Pharma, Tokyo, Japan). The patient was not supplemented with vitamin D and calcium.

At follow-up, 2 weeks after injury, she was still complaining of intolerable back pain during activity, and the VKA was 6°, showing significant instability of the affected vertebra (Figure 1(b)). Because the patient was living alone and no one could take care of her, she was hospitalized to allow suitable rest. The patient was restricted to complete bed rest for 3 weeks and, thereafter, was gradually allowed to walk with aids while wearing the TLSO. Back pain subsided by 2 months after injury. Spinal X-ray obtained 2 months after injury showed vertebral stabilization with consolidation of the posterior wall, and VKA was measured as 8° (Figure 1(c)), showing correction of 12° in the affected vertebra compared to baseline and successfully union due to teriparatide treatment and hospitalization. The patient was discharged 2.5 months after injury. TLSO was applied 6 months after injury. As of the latest follow-up at 7 months after injury, VKA remained at the corrected position (8°) although the anterior vertebral cleft remained (Figure 1(d)).

## 3. Discussion

The natural history of osteoporotic vertebral fractures usually leads to spontaneous consolidation of the deformed vertebral body, but in some cases instability remains after fracture and can be a cause of persistent pain [5]. In patients with nonunion showing instability, surgical interventions can provide stability and pain relief. In addition, correction of the deformity has until now been achieved only with surgical treatment. A review article reported the potential of kyphosis reduction with kyphoplasty using polymethylmethacrylate (PMMA) cement in fresh fractures, offering height restoration of 0–90% and a mean absolute correction of kyphotic angle of 8.5° [5]. The angle of correction obtained in the present case (12°) was comparable to or better than the mean angle obtained with kyphoplasty using PMMA.

Teriparatide is a powerful antiosteoporotic agent, and randomized clinical trials have shown that teriparatide significantly increased bone mineral density (BMD) in the lumbar spine by 6.7–13.4% from baseline [6–8] and prevented new vertebral fractures with a relative risk reduction of 65–84% compared to placebo controls [7–9]. In addition to fracture prevention, since teriparatide has potent bone-forming effects [10, 11], this agent has been expected to enhance fracture healing in patients with delayed healing or nonunion [12]. A randomized clinical trial suggested a role for teriparatide in accelerating healing for fractures of the distal radius in postmenopausal women [13]. In our recent study, teriparatide treatment significantly shortened the time to fracture healing and reduced rates of delayed healing or nonunion after bisphosphonate-associated atypical femoral fractures [14].

We have recently compared the progression of vertebral collapse after osteoporotic fresh vertebral fracture among the groups treated with teriparatide (20 *μ*g/day or 56.5 *μ*g/week) and risedronate (17.5 mg/week) and untreated control group [3]. All patients were hospitalized and followed up for 12 weeks after fracture. Progression of vertebral collapse was significantly prevented by teriparatide (*p* < 0.05), but not by risedronate, compared to untreated control [3]. These results showed that teriparatide, but not risedronate, is promising for preventing the progression of vertebral collapse after osteoporotic vertebral fractures through potent bone-forming effects allowing early bone healing. Furthermore, in the present case, a fractured vertebra with an intravertebral cleft was correctively healed with once-weekly teriparatide without surgical intervention. While the pathogenesis underlying the intravertebral cleft sign is not completely understood, the sign is most suggestive of ischemic necrosis of bone [15]. Osteonecrosis of the fractured vertebrae often results in pseudoarthrosis requiring surgery to address symptomatic intravertebral instability [2]. In the present case, the patient initially experienced intolerable pain with intravertebral instability but achieved successful corrective healing with teriparatide. Teriparatide may have the potential to heal unstable vertebral fractures and achieve morphological correction.

In this case, baseline serum 25-hydroxyvitamin D status was not evaluated and the patient was not supplemented with vitamin D. Because vitamin D insufficiency is known to exert negative influences on BMD [16], the sufficiency of vitamin D may also be an important factor in the healing of fractures. Although recent studies have shown that significant increases in BMD can be achieved in patients receiving teriparatide regardless of baseline vitamin D levels [17], teriparatide effects on healing of fractures under the influence of vitamin D levels should be clarified.

In conclusion, with our experience in treating unstable vertebral fracture in an elderly woman, teriparatide in combination with hospitalized rest and TLSO may have the potential to heal unstable vertebral fractures in a corrective shape without surgical intervention.
